# Evaluation of osteoblastic activity in extraction sockets treated with platelet-rich fibrin

**DOI:** 10.4317/medoral.19999

**Published:** 2014-12-05

**Authors:** Ozgur Baslarli, Celal Tumer, Omer Ugur, Betul Vatankulu

**Affiliations:** 1 Doctor in Dentistry, PhD, Hacettepe University Faculty of Dentistry, Department of Oral Surgery, Ankara, Turkey; 2 Private Practice, Prof. Dr., retaired from Hacettepe University Faculty of Dentistry, Department of Oral Surgery, Ankara, Turkey; 3 Doctor in Medicine, Prof. Dr., Hacettepe University Faculty of Medicine, Department of Nucleer Medicine, Ankara, Turkey; 4 Doctor in Medicine, PhD, Istanbul University Faculty of Medicine, Department of Nucleer Medicine, Istanbul, Turkey

## Abstract

Objective: The aim of this study was to determine whether the use of platelet rich fibrin (PRF) improved the healing of extraction sockets. Study Design: A total of 20 patients with bilateral soft tissue impacted mandibular third molars were included in this study. The left and right third molars were extracted during the same session. Subsequently, the PRF membrane was randomly administered to one of the extraction sockets, whereas the contra lateral sockets were left without treatment. On postoperative 30. and 90. days, panoramic images and bone scintigrams were taken to evaluate the bone healing between PRF-treated and non-PRF-treated sockets. Also, periodontal evaluation was performed in the same control sessions. Dependent group t test for paired samples was used for statistical analysis. Results: The average increase in technetium-99m methylene diphosphonate uptake as an indication of enhanced bone healing did not differ significantly between PRF-treated and non-PRF-treated sockets 30 and 90 days post operatively. Radio opacity that can show the bone healing on panoramic images were measured by Image J programmer and they did not differ significantly. Also periodontal values did not differ significantly. Conclusions: PRF might not lead to enhanced bone healing in impacted mandibular third molar extraction sockets 30 and 90 days after surgery. It is thought that PRF has the potential characteristics of an autologous fibrin matrix and can accelerate the healing. To better understand the effects of PRF on healing, further research is warranted with larger sample sizes.

** Key words:**PRF, scintigraphy, healing, extraction sockets.

## Introduction

Complex tissue remodeling requires the coordination of various physiological processes, which involve molecular signals that are mediated primarily by cytokines and growth factors (1). Platelets contain various growth factors and cytokines that play a key role in inflammation and bone healing (2). Given these physiological traits, the use of platelet concentrates has become increasingly popular during last 15 years. The initial development of platelet concentrate technology in 1996 offered simplified and optimized production protocols for a new type of fibrin-adhesive concentrated platelet-rich plasma (cPRP) (3). After 5 years, a second generation platelet concentrate named platelet rich fibrin (PRF) was introduced in France (3).

The advantages of PRF over PRP include ease of preparation/application, minimal expense, and lack of biochemical modification (i.e., no bovine thrombin or anticoagulant is required). PRF predominantly consists of a fibrin matrix rich in platelet, leukocyte cytokines and growth factors (4). In the field of oral and maxillofacial surgery, PRF was first used with dental implantology.

To reduce alveolar bone dimensional changes, several techniques aimed at enhancing the regeneration process in the extraction socket have been adopted, such as autogenous bone grafts or bone substitutes to fill sockets; guided bone regeneration (GBR) with re sorbable or non-re sorbable barriers; and the use of various bone promoting molecules such as enamel matrix derivative, recombinant growth and differentiation factors, and autologous platelet concentrates. These techniques have been used alone or in combination by clinicians in their search for the optimal socket preservation method (5).

The use of platelet concentrates has been proposed as an aid for enhancing the regeneration of osseous and epithelial tissues in oral surgery (5). Several studies have suggested that platelet concentrates, especially PRF, may stimulate osseous and soft tissue regeneration while also reducing inflammation, pain and unwanted side effects (6,7).

The aim of this study was to determine whether the use of PRF improved the healing of extraction sockets.

## Patient and Methods

The inclusion criteria of patients in the study was presence of bilateral soft tissue impacted third molars that were positioned in a vertical or mildly mesio-angular direction. Patients with a medical history, patients who had smoking habits and patients with mandibular third molars requiring bone removal during surgery were excluded from this study. Patients who were referred to the Department of OMFS, Faculty of Dentistry, Hacettepe University (Ankara, Turkey) with pain or discomfort in the mandibular third molar region were examined for their possible recruitment in the study. In accordance with the inclusion and exclusion criteria, 47 of 96 patients underwent further radiographic and oral examinations. Twenty patients (7 males and 13 females, age range = 19-34 years, average age = 23.9 years) were included in this study.

The protocol was reviewed by the Ethics Committee of Hacettepe University and is in compliance with the Helsinki Declaration. Each subject signed a detailed informed consent form.

Patients underwent surgical treatment in accordance with the rules of antisepsis and asepsis. All treatments were performed by the same experienced surgeon. Prior to the extractions, 9 ml of venous blood was collected from each patient and was placed in anticoagulant-free glass tubes. Tubes were transferred to a centrifuge device and centrifuged for 10 minutes at 3000 rpm. Following centrifugation, PRF was dissected approximately 2 mm below its connection to the red corpuscle beneath to include remaining platelets, which have been proposed to localize below the junction between PRF and the red corpuscle. Then PRF was squeezed between gauzes to transform into a membrane.

Mandibular and buccal blocks were administered using articaine containing 1:200,000 epinephrine. Before the surgical procedure, pocket depths of the neighboring teeth (e.g., tooth numbers 37 and 47) were measured from six points (e.g., mid, mesial and distal parts of the buccal and lingual aspects) using Michigan periodontal probe. After achieving anesthesia, sulcular and vertical incisions were made around the tooth to be extracted before full thickness flaps were reflected. The right and left third molars were carefully extracted during the same session. Subsequently, the PRF membrane was administered to one of the extraction sockets, which was chosen randomly. The surgical incisions were primarily closed with 3.0 silk sutures. Amoxicillin (2 x 1000 mg) and naproxen sodium (3 x 550 mg) were administered for 5 days post operatively for each patient. Sutures were removed on postoperative day 7 in all cases.

Postoperative panoramic images and bone scintigrams were taken and periodontal evaluation was performed on postoperative days 30 and 90.

- Scintigraphic Study

**Figure 1:**
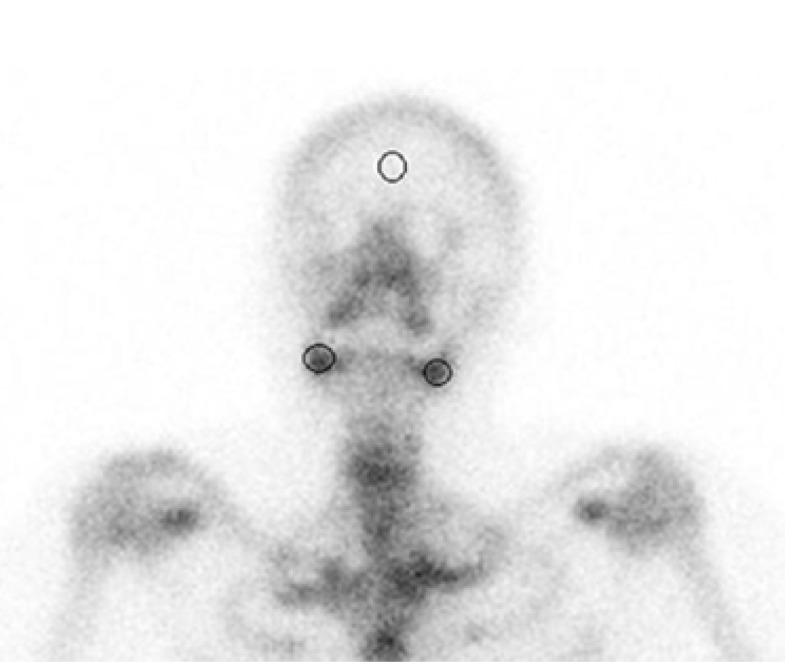
Regions of interest drawn on the scintigram to indicate extraction socket regions and the frontal bone of calvarium.

On postoperative days 30 and 90, bone scans with technetium (TC) 99m methylene diphosphonate were performed for each patient. The intravenous injection of 555 MBq of Tc 99m methylene diphosphonate was performed; static images were acquired after 3 hours of injection. Circles were drawn on the operative sites to indicate the regions of interest, whereas circles of the same sizes were drawn in the contra lateral regions (Fig. 1). Peak values of the given radio pharmaceuticals were calculated using dynamic scans ( Table 1). All of the scintigraphic assessments were conducted by the same nuclear medicine physician, who was blinded to the study.

**Table 1:**
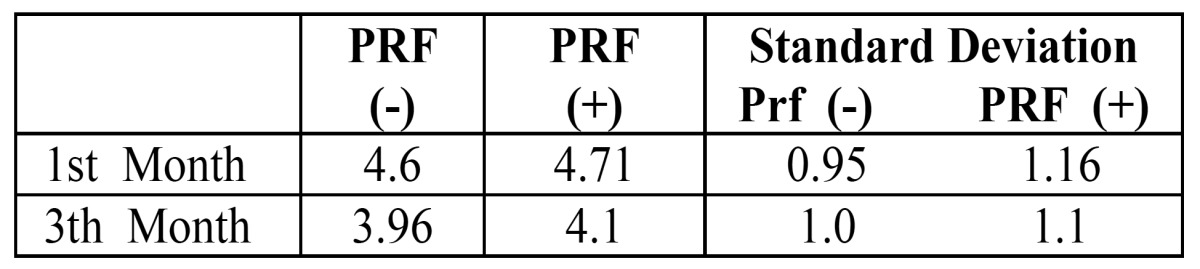
The averages values of technetium-99 methylene diphosphonate uptake in PRF treated and non-PRF treated sockets in first and third months.

- Panoramic Images Study

In the same session, panoramic images were taken for each patient. Circles were drawn on the operative sites to indicate the regions of interest, whereas circles of the same size were drawn in the contralateral regions. Peak mean gray values (MGV) were calculated using the Image J© software program. All radiographic assessments were conducted by the same radiologist, who was blinded to the study.

- Periodontal Pocket Depth Measurement

Before the surgery, periodontal pocket depths of the neighboring teeth (e.g., tooth numbers 37 and 47) were measured from six points (i.e., mid, mesial and distal parts of the buccal and lingual aspects) using Michigan periodontal sond. In the same follow-up session, periodontal pocket depths of the neighboring teeth were again measured. A probing depth of at least 4 mm was considered an indicator for periodontal pathology.

- Statistical Analysis 

Statistical analysis was performed by the same biostatistician from the Department of Biostatistics, Faculty of Medicine, Hacettepe University (Ankara, Turkey). A dependent group t test for paired samples was used for each patient to compare healing improvement between extractions sockets treated with and without PRF.

## Results

All patients who participated in this study attended the 30- and 90-day appointments.

Two patients developed postoperative secondary infections. One patient developed an infection two months after the procedure, with pain and a 5-mm pocket on the distal surface of the second molar. The other patient displayed pain, swelling and an 8-mm pocket on the distal surface of the second molar tooth three weeks post operatively. Pus was also noted. Both infected areas were in the non-PRF treated regions. However, the periodontal probing depths of the second molars between PRF treated and non-PRF treated sockets did not show statistically significant differences (*P* < .01) on the 30- and 90-day postoperative visits.

**Figure 2:**
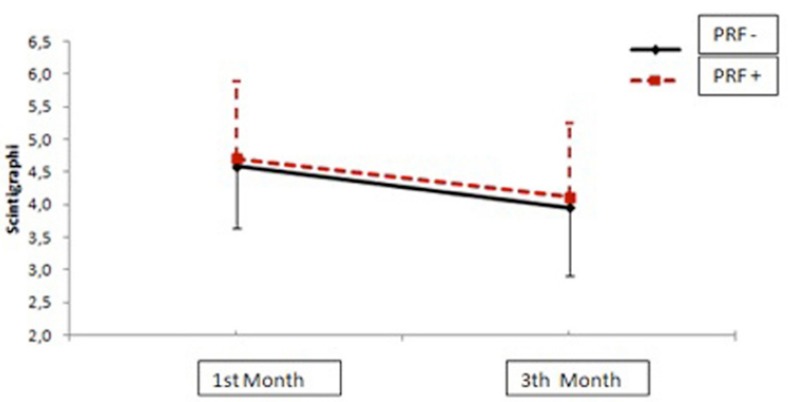
The averages of Tc-99 methylene diphosphonate uptake in PRF treated and non-PRF treated sockets in the first and third months.

The averages of technetium-99 methylene diphosphonate uptake in PRF treated and non-PRF treated sockets were similar and not statistically significant (Fig. 2) on both postoperative visits.

**Figure 3:**
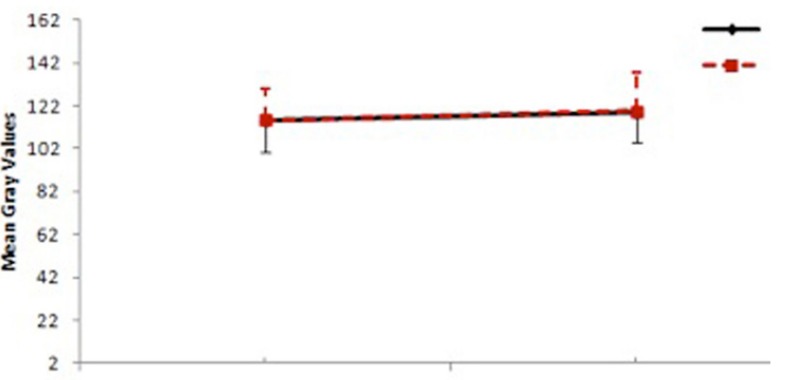
The average mean gray value scores of the bone obtained from panoramic radiographs in the first and third months.

When panoramic films were analyzed using Image J©, the average mean gray value scores of the bone in the extraction areas with or without PRF were similar, without statistically significant differences (Fig. 3), on both postoperative visits.

## Discussion

This study analyzed the effects of PRF in extraction sockets 30 and 90 days after surgery. Bone scintigrams were used to assess bone mineralization and osteoblastic activity in the extraction sockets. A correlation was observed between osteoblastic cell activation and absorption of technetium-99m. In contrast to radiographic and histological techniques, early bone mineralization of the extraction sockets was determined using scintigraphic images. In a pilot study (8) , Bambini et al. investigated the effects of immediate prosthetic loading on peri-implant osteoblastic activity using bone scintigraphy 30 and 90 days after surgery. The scores between the two scintigrams showed that peri-implant osteoblastic activity was very high in the first month, before declining over subsequent months.

Bone scintigraphy is an expensive method and loads the patient with radionuclear energy for 24 hours. However, a nuclear bone scintigraphy is a functional test: it measures an aspect of bone metabolism or bone remodeling, which most other imaging techniques (magnetic resonance imaging, computed tomography) cannot. When compared with the other methods it is less expensive and it has no permanent or temporary side effects.

For determining osteoblastic activity, bone scintigraphs were used in several studies. In a study Gurbuzer *et al*. (7) evaluated the effect of PRF on early bone healing using bone scintigraphy in third molar extraction sockets. In an another study, Gulaldi *et al*. (9) evaluated defect sites treated with various grafting materials using scintigraphic images. In light of these studies bone scintigraphy was used to determine the osteoblastic activity in extraction sockets treated with or without PRF. As increased technetium-99 methylene diphosphonate uptake indicates areas of new bone formation, bone scintigraphy is an appropriate method for investigating osteoblastic activity within healing extraction sockets. Because of its superior characteristics in detecting short-term osteoblastic activity and bone healing levels, bone scintigraphy was utilized in this study as the primary assessment method.

Panoramic films were obtained in addition to scintigraphic images. Radiopaque changes in the extraction sockets on panoramic films were assessed with numerical mean gray values using the Image J© program. Mean gray value is the sum of the gray values of all the pixels in the selection divided by the number of pixels. Mean gray value gives numerical values about radiopacity, hereby bone healing. Zhao et al. (10) and Chiapasco *et al*. (11) both evaluated bone healing using mean gray values. Because of the low radiation exposure and high patient acceptance, panoramic films are the most frequently used type of radiographs for the evaluation of hard tissues in dental practice. However, panoramic films can be preliminary screening tool for measuring bone density. Soft tissue thickness can affect overall density of the panoramic film (12). So in this study, panoramic films were obtained as a supportive method to bone scintigraphy.

Platelet rich fibrin (PRF) was first described by Choukroun *et al*. (3) as a second generation platelet rich concentrate that acts as fibrin membranes enriched with platelets and growth factors. PRF induces cell migration, proliferation and cicatrization (3,13). PRF is thought to help with wound healing, expedite angiogenesis and epithelialization, regulate inflammation and release growth factors (e.g., platelet derived growth factor (PDGF), transforming growth factor (TGF), vascular endothelial growth factor (VEGF), and insulin-like growth factor (ILGF) (13) as well as cytokines (e.g., (interleukin (IL)_IL-1β, IL-6, IL4, and tumor necrosis factor-α) (14). The development of platelet concentrate technologies offers simplified and optimized production protocols. According to these protocols, PRP is considered first generation autogenous fibrin adhesive and PRF is the second generation (3); thus, it is different than the first generation fibrin-adhesive platelet rich plasma (PRP). PRF is essentially centrifuged blood without any additions (15); it is obtained via slow polymerization that effects the 3-dimensional organization of the fibrin network. This type of organization has equilateral junctions, which makes the fibrin membrane flexible, elastic and strong (15).

Although PRF has gained popularity in dental settings in recent years, only a few studies in the literature have investigated its healing effects. In this study, the healing potential of bone was investigated with by comparing PRF-treated and non-PRF-treated extraction sockets. In conclusion, PRF-treated extraction sockets did not demonstrate any differences than non-PRF-treated extraction sockets post operatively after 4 and 12 weeks.

Various reasons may explain why PRF failed to increase healing in the extraction sockets. Although growth factors can stimulate osteoblastic proliferation and chemotaxis, the effects of different combinations of growth factors and their actions may vary depending on the cell population and culture conditions (16). The reported actions of these factors vary from one cell population to another or within the same cell population under different culture conditions (17). In addition, certain combinations of growth factors may have synergistic (18) or antagonistic effects or both (19,20). Karsperk *et al*. (18) noted that FGF, TGF-β and PDGF can increase DNA synthesis, whereas they can decrease alkaline phosphatase (ALP) synthesis when used together. Giannobile *et al*. (19) evaluated combinations of IGF-1, PDGF, TGF- β1 and FGF on bone remodeling and differentiation and found that combining IGF-1 and other growth factors increases mitogenic activity and protein synthesis of osteoblasts, while decreasing ALP synthesis. ALP synthesis and bone mineralization have a positive correlation. In this study, the effects of these growth factors (IGF-1, PDGF, TGF-β1 and FGF) may have decreased ALP synthesis with an antagonistic effect, thereby decreasing bone mineralization. Decreased mineralization may have affected the emission ofTc 99m by the osteoblasts.

Dohan *et al*. (20) analyzed in vitro effects of PRF on human bone mesenchymal stem cells (BMSC). They cultivated BMSCs with or without a PRF membrane originating from the same donor as the cells, in both proliferative and osteoblastic differentiation conditions. In another group they used 2 PRF membranes to measure the dose-dependent effect. Cell counts, cytotoxicity tests, ALP activity, Von Kossa staining and mineralization nodules were counted. The authors found that PRF significantly stimulated BMSC proliferation and differentiation, with a dose-dependent effect.

In this study, venous blood was collected in a single 9 ml glass tube before PRF was prepared. Although such tubes have generally been utilized in the literature, the PRF dose may not have been sufficient in terms of osteoblastic activity at both postoperative visits. In an another in-vitro study, a larger amount of PRF should be used to learn whether it is dose-dependent or not.

We also measured pocket depths of the mandibular second molars at both follow-up appointments. Similarly, Kan *et al*. (21) measured the pocket depths in 158 patients after mandibular third molar extractions 6-36 months after surgery and found that 67% of the neighboring mandibular second molars had pocket depths of >5 mm.

In another study, Sammartino *et al*. (22) extracted bilateral mandibular third molars, after which they applied PRP to only one extraction socket. Pocket depths of neighboring teeth were healthier in the PRP-treated sockets, thus highlighting the potential effect of PRP on periodontal healing.

In this study, 2 patients displayed increased pocket depths and pain around mandibular second molar extraction sockets that were treated without PRF. One of the pockets was detected in the first month, whereas the other pocket was found in the third month. Nevertheless, the inflammations in the non-PRF-treated group were not statistically significant. To better assess the potential effects of PRF on periodontal healing, future studies are warranted with larger sample sizes.

In conclusion, this study was performed to illuminate the potential effects of PRF on extraction sockets and tissue healing. However, the findings failed to indicate any significant effects of PRF for the healing period in mandibular third molar extraction sockets.
